# Ireland’s medical brain drain: migration intentions of Irish medical students

**DOI:** 10.1186/s12960-015-0003-9

**Published:** 2015-03-12

**Authors:** Pishoy Gouda, Kevin Kitt, David S Evans, Deirdre Goggin, Deirdre McGrath, Jason Last, Martina Hennessy, Richard Arnett, Siun O’Flynn, Fidelma Dunne, Diarmuid O’Donovan

**Affiliations:** National University of Ireland, Galway, Ireland; Department of Public Health, Merlin Park Hospital, HSE West, Galway, Ireland; University of Limerick, Limerick, Ireland; University College Dublin, Dublin, Ireland; Trinity College Dublin, Dublin, Ireland; Royal College of Surgeons in Ireland, Dublin, Ireland; University College Cork, Cork, Ireland

**Keywords:** Medical students, Emigration and immigration, Human resources

## Abstract

**Background:**

To provide the optimum level of healthcare, it is important that the supply of well-trained doctors meets the demand. However, despite many initiatives, Ireland continues to have a shortfall of physicians, which has been projected to persist. Our study aimed to investigate the migration intentions of Irish medical students and identify the factors that influence their decisions in order to design appropriate interventions to sustain the supply of trained doctors in order to maintain a viable medical system.

**Methods:**

An online cross-sectional survey was undertaken of all Irish medical students studying in the Republic of Ireland. The survey included nominal, ordinal, and scale items to determine migration intentions, factors influencing their decisions, and understanding of the Irish healthcare system.

**Results:**

A total of 2 273 medical students responded (37% response rate), of whom 1 519 were classified as Irish medical students (having completed secondary school in Ireland). Of these, 88% indicated they were either definitely migrating or contemplating migrating following graduation or completion of the pre-registration intern year. Forty percent expressed an intention of returning to Ireland within 5 years. The factors most influencing their decision to leave were career opportunities (85%), working conditions (83%), and lifestyle (80%).

**Conclusion:**

The migration intentions expressed in this study predict an immediate and severe threat to the sustainability of the Irish healthcare service. Urgent interventions such as providing information about career options and specialty training pathways are required. These must begin in the undergraduate phase and continue in postgraduate training and are needed to retain medical school graduates.

## Background

To provide an effective healthcare system it is critical that there is an adequate supply of highly trained medical professionals [1]. However, shortages have been experienced in several developed countries [1-3], leading to policy initiatives to address these shortages. In the U.K., doctor’s salaries have been increased and the “Improving Working Lives Standard” has been implemented, while also increasing international recruitment initiatives [4]. Australia and New Zealand have employed similar interventions in addition to increasing the number of domestic medical students [5]. In Ireland, there has been an expansion in medical undergraduate training capacity [6], government investment in undergraduate and postgraduate education and training [6-8], and increased recruitment of doctors from overseas [9]. Despite these initiatives, Ireland continues to have a shortfall of physicians which has been projected to continue [10-13]. It is currently unclear why current initiatives in Ireland have been ineffective in preventing physician shortages.

The difficulty of retaining qualified Irish doctors within the Irish healthcare system has been highlighted in four recent surveys. The first demonstrated that 80% of non-consultant hospital doctors (NCHDs) were contemplating emigration [14]. Another showed that 63% of NCHDs were considering working abroad in the next 3 years [12]. A survey of interns showed only 53% intended to continue working in Ireland upon completion of their intern year [7]. In a survey of 186 final-year Irish medical students, it was demonstrated that 66% did not intend to work in an Irish hospital 1 year postgraduation [15].

A key difficulty is the migration of Irish trained doctors once they have qualified or completed their pre-registration (intern) year. While Ireland has the highest level of physician emigration in Europe and the second highest in the world [16,17], the reasons why physicians emigrate are unclear. Simoens and Hurst [1] suggest that emigration among OECD medical professionals may be due to a lack of clarity regarding career pathways, long hours, and lack of support in receiving advanced training. These factors have been shown to affect migration patterns in developing countries [1,18,19].

While medical migration can produce many positive effects such as increased international collaboration and return of nationals with foreign-derived expertise and education [20], the short-term effect is a lack of qualified personnel. Although there has been one study on final-year medical students [15], the existing literature on medical migration focuses on medical graduates. In addition, it is unclear from the literature whether the intention to migrate is formed earlier during undergraduate training or indeed prior to the commencement of training (with students choosing medicine as it provides them with the opportunity to migrate). This has implications for the timing of initiatives to address migration. The relatively small sample sizes of current Irish literature (ranging from 87 to 178 respondents) also limit the degree to which generalisations can be made in terms of national patterns. This study aimed to investigate the migration intentions of medical students in Ireland, and the factors which influence their decisions.

## Methods

We conducted a cross-sectional survey of all Irish medical students attending Irish medical schools. Irish students were selected due to the current high level of physician emigration [16,17]. In addition, European working laws make it difficult for non-Irish students to be employed on a permanent basis postregistration [21]. The survey was administered via an online survey tool (Survey Monkey v. 28 March 2012 http://www.surveymonkey.net). All registered medical students receive an e-mail address. Each of the six medical schools in Ireland (National University of Ireland, Galway, University of Limerick, University College Cork, Royal College of Surgeons of Ireland, University College Dublin and Trinity College Dublin) agreed to contact all registered medical students by e-mail to inform them about the study and provide them with the URL link to access the survey. An incentive was offered to those who participated (entered into a draw for a tablet computer). A reminder e-mail was sent after 2 weeks. The survey remained open for approximately 21 days.

The questionnaire aimed to elicit intentions to migrate after graduation, perceptions of factors which influence migration intentions, understanding of postgraduate training in Ireland, and understanding of the Irish healthcare system. It contained a combination of nominal, ordinal, and scale items. Respondents were initially asked if they had completed secondary school in Ireland. Those who responded “yes” were defined as Irish and became the focus of the study, while those who responded “no” were excluded from the analysis.

Migration intentions were assessed by asking the question, “Which of the following describes you best?” Response choices were (1) I am definitely going abroad for work/further training after graduation or intern year, (2) I am contemplating going abroad for work/further training after graduation or intern year, (3) I have contemplated but decided against going abroad for work/further training after graduation or intern year, (4) I am definitely not going abroad for work/further training after graduation or intern year, and (5) I am unsure about my plans after graduation and intern year. Those definitely or contemplating going abroad were asked, “Which of the following would influence your decision to go abroad for work/further training? (Select all applicable)”. Response choices were (1) pay, (2) career opportunities, (3) family, (4) lifestyle, (5) independence, (6) obligation, (7) working conditions in Ireland, (8) standard of training, (9) debt, and (10) other (specify). To assess understanding of postgraduate training and the Irish healthcare system, respondents were asked to rate the statements, “I understand what training is involved after the intern year” and “Overall I have a good understanding of how the Irish healthcare system works.” Response choices were (1) strongly agree, (2) agree, (3) neither agree nor disagree, (4) disagree, (5) strongly disagree, and (6) not applicable. We also collected sociodemographic information such as age, sex, year of study, course duration, location of secondary school completion, and previous third-level qualifications. All questions were required to be completed before submission would be accepted.

As the duration of the medical courses varies between 4 and 6 years (6-year course is divided into one premedical year and five medical teaching years), senior medical students were defined as medical students in their fourth or fifth year of study, intermediate medical students were defined as medical students in their second or third year of study, while junior medical students were defined as medical students in their premedical/foundation year or first year.

Pearson’s Chi square and Independent *T* tests were utilised to determine the significance of differences in responses to questions by sex, age, and migration intentions. Multinomial logistic regression was undertaken to determine if stage of training is associated with migration intentions, controlling for age and sex. Those definitely going abroad were defined as the reference category (excluding those unsure about migration plans) with degree stage as the predictor variable. SPSS v.20 was used for data analysis. Ethical approval was granted by the Research Ethics Committee at the National University of Ireland, Galway.

## Results

### Response rates

The survey was sent to all 6 180 medical students enrolled in Irish medical schools (2012–2013 academic year), of whom 37% (*n* = 2 273) responded. The response rate varied between medical schools, ranging from 20% to 54%. Of those who responded, 67% (*n* = 1 519) had completed secondary school in Ireland, 5% (*n* = 121) in another EU country, and 28% (*n* = 633) in a non-EU country. The remainder of the analysis presented relates to the subgroup that completed secondary school in Ireland, which for our study’s purposes was identified as Irish medical students.

### Study population

The profile of respondents is given in Table 1. Overall, 57% (*n* = 865) of respondents were female, with 29% (*n* = 447) aged 18–20, 42% (*n* = 636) aged 21–23, and 27% (*n* = 406) over 23 years of age. A quarter (*n* = 372) had a degree before entering medical school. Junior, intermediate, and senior medical students accounted for 27% (*n* = 405), 43% (*n* = 658), and 30% (*n* = 456) of respondents, respectively. Based on data provided by the Health Education Authority of Ireland^a^, the study population is broadly representative of the overall population of medical students in terms of age (e.g. 28% of the overall population are aged 18–20, 39% aged 21–23, and 33% aged over 23 years), although the study population does contain a larger proportion of females (57% compared to 51% for the overall population).

**Table 1:** **Profile of respondents**

**Profile**	**No**	**%**
Age		
18–20	447	29.4
21–23	636	41.9
Over 23	436	28.7
Sex		
Male	654	43.1
Female	865	56.9
Degree before entering medical school		
Yes	372	24.5
No	1 147	75.5
Stage in degree programme		
Junior (premedical/foundation year and first year)	405	26.7
Intermediate (second and third year)	658	43.3
Senior (fourth and fifth year)	456	30.0

### Migration intentions

In this study, 88% (*n* = 1 332) of Irish medical students stated that they were either definitely (34% *n* = 522) or contemplating (53% *n* = 810) going abroad after graduation or after the pre-registration intern year. The remainder were either unsure of their plans (9% *n* = 139) or had decided they definitely were not going abroad (3% *n* = 48). There were no significant differences between the sexes in intentions to migrate (*χ*^2^ = 7.388, df = 10, *p* = 0.181).

**Figure 1:**
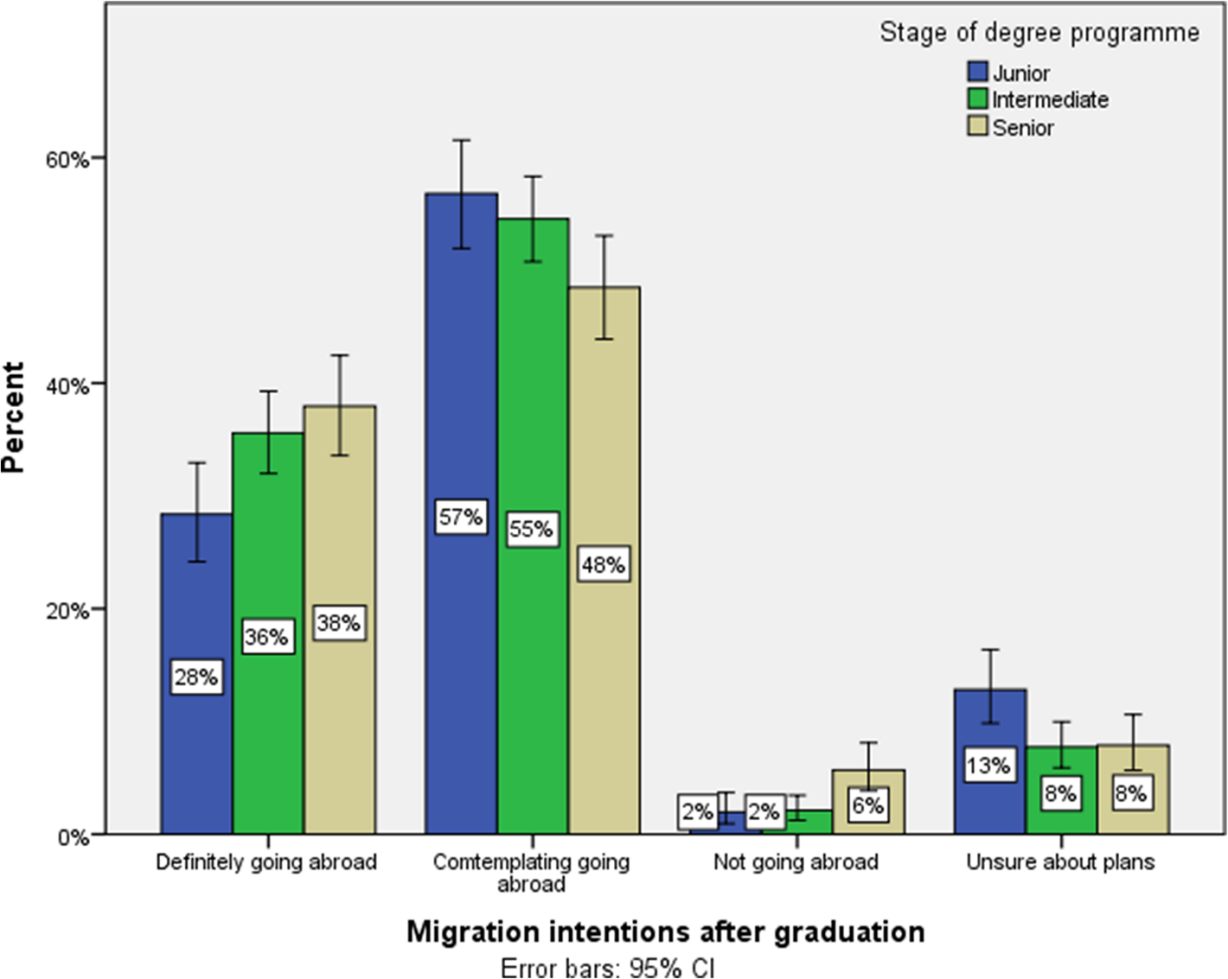
**Migration plans by stage of degree programme (with 95% confidence intervals).**

Figure 1 shows migration intentions by the respondents’ stage in their degree programme. The proportion definitely intending to go abroad increased from 28% (*n* = 115) at the junior stage to 36% (*n* = 234) at the intermediate stage to 38% (*n* = 173) at the senior stage of training. The proportion contemplating going abroad reduced from 57% (*n* = 230) at the junior stage to 55% (*n* = 359) at the intermediate stage to 49% (*n* = 221) at the senior stage of training. Multinomial logistic regression (Table 2) found that there was a significant association between migration intentions and stage of training (*χ*^2^ = 17.292, df = 4, *p* = 0.002). After controlling for age and gender, those definitely going abroad were more likely to be second- and third-year students (odds ratio [OR] = 2.528, *p* = 0.016) than those not going abroad. In addition, those contemplating going abroad are more likely to be pre- and first-year students (OR = 3.573, *p* = 0.009) and second- and third-year students (OR = 3.123, *p* = 0.003) than those definitely not going abroad.

**Table 2:** **Multinomial logistic regression of stage of training by migration intentions (controlling for age and sex)**

**Migration intentions**		**Sig.**	**OR**	**95% confidence interval for OR** ^**a**^	
				**Lower bound**	**Upper bound**
Definitely going abroad	Intercept	0.000			
	Age^b^ (18–21 and over 21 years)	0.969	1.015	0.474	2.174
	Sex^c^	0.595	0.850	0.466	1.550
	Stage of training^d^				
	Junior	0.115	2.188	0.826	5.794
	Intermediate	0.016	2.528	1.192	5.362
Contemplating going abroad	Intercept	0.000			
	Age^b^ (18–21 and over 21 years)	0.830	1.086	0.512	2.304
	Sex^c^	0.904	1.037	0.573	1.879
	Stage of training^d^				
	Junior	0.009	3.573	1.369	9.325
	Intermediate	0.003	3.123	1.485	6.565

^a^Reference category: definitely not going abroad.

^b^Reference category: 18–21 years.

^c^Reference category: male.

^d^Reference category: senior stage of training.

### Factors influencing migration intentions

**Figure 2:**
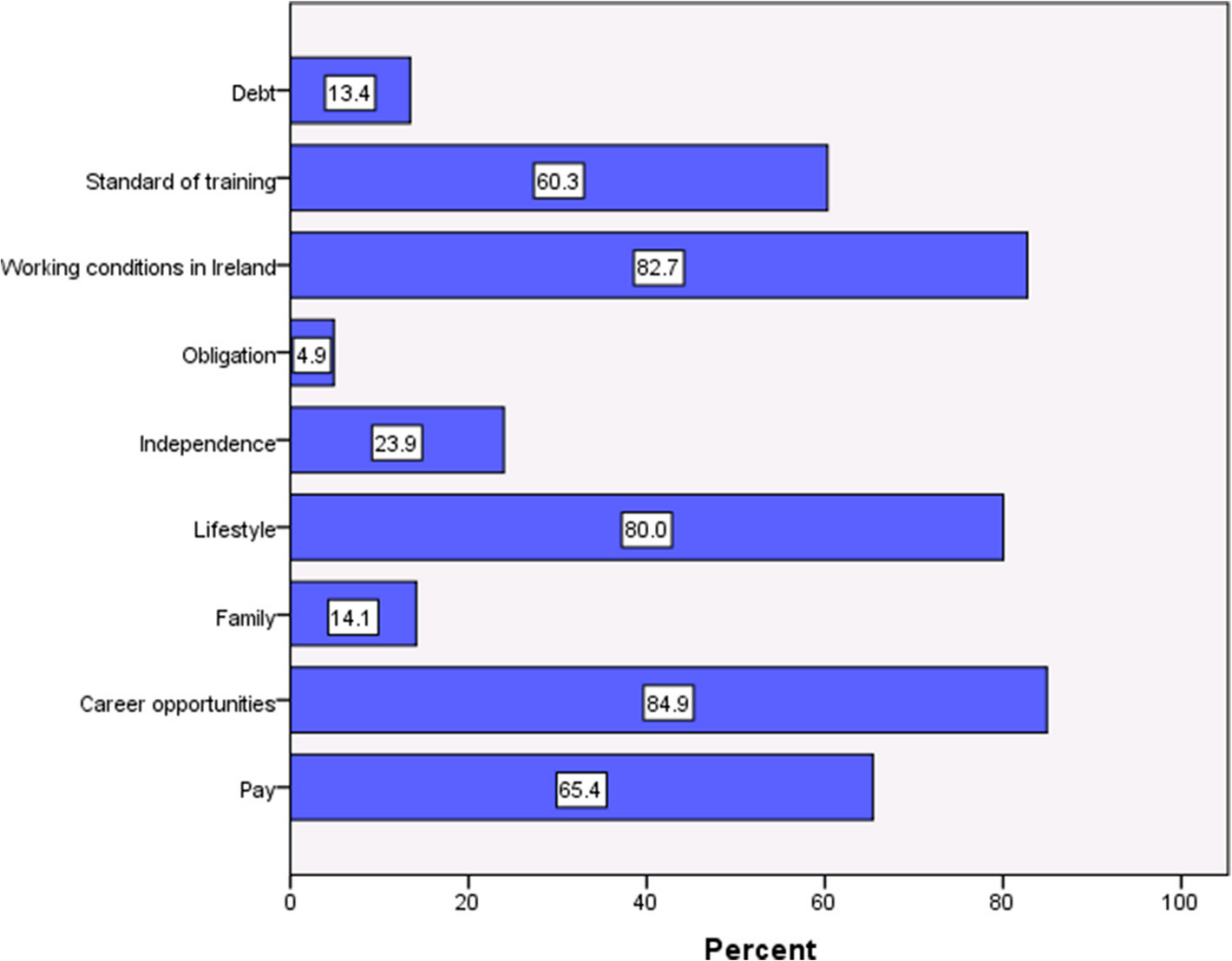
**Push factors of students contemplating or definitely going abroad.**

Those who were definitely or contemplating going abroad were asked for the reasons influencing their decision (Figure 2). The main reasons given were career opportunities (85%), working conditions in Ireland (83% *n* = 1 102), lifestyle (80% *n* = 1 066), pay (65% *n* = 871), and the standard of training (60% *n* = 803). There were no significant differences in reasons given by sex (*p* > 0.05). The proportion citing salary (*χ*^2^ = 10.122, df = 2, *p* = 0.006), working conditions in Ireland (*χ*^2^ = 38.659, df = 2, *p* = 0.000), the standard of training (*χ*^2^ = 11.466, df = 2, *p* = 0.003), and debt (*χ*^2^ = 196.934, df = 2, *p* = 0.000) as reasons was significantly greater in the older age groups (over 23 compared to 21–23 and 18–20). Conversely, a significantly lower proportion of the older age groups reported independence as a reason (*χ*^2^ = 32.768, df = 2, *p* = 0.000). The 48 respondents definitely not going abroad primarily cited family (81%, *n* = 39) as an issue influencing their decision not to migrate.

### Understanding of postgraduate training and the Irish healthcare system

Respondents were asked to rate their understanding of the training involved after the intern year and also their understanding of how the Irish healthcare system works. Table 3 shows that for those that had decided their future migration plans (excluding those unsure of future plans, *n* = 139), 7% (*n* = 78) agreed that they understood the training involved after the intern year, with 59% (*n* = 687) disagreeing or strongly disagreeing. A larger proportion of those not going abroad agreed that they understood the training involved compared to those definitely or contemplating migrating (19% compared to 6%). This pattern is statistically significant (Independent *T* test: *t* = 2.961, *p* = 0.003). There were no significant differences in rating understanding of the training by sex (Independent *T* test, *t* = 1.068, *p* = 0.286). A larger proportion of those aged under 21 years (71%) disagreed or strongly disagreed that they understood the training involved after the intern year compared to those aged over 21 years (50%). This pattern was statistically significant (Independent *T* test, *t* = 9.048, *p* = 0.001).

**Table 3:** **Rating of understanding of postgraduate training and the Irish healthcare system by plans to go abroad (1 = strongly agree, 5 = strongly disagree)**

	**Plans to go abroad** ^**a**^					
	**Definitely or contemplating**		**Not going abroad**		**Total**	
	**No.**	**%**	**No.**	**%**	**No.**	**%**
I understand what training is involved after the intern year^b^						
Strongly agree						
Agree	70	6.3	8	19.0	78	6.7
Neither	378	33.8	17	40.5	395	34.1
Disagree	218	19.5	6	14.3	224	19.3
Strongly disagree	452	40.4	11	26.2	463	39.9
Mean	3.94		3.48		3.93	
Overall I have a good understanding of how the Irish healthcare system works^c^						
Strongly agree						
Agree	85	7.5	8	18.2	93	7.9
Neither	455	40.2	20	45.5	475	40.4
Disagree	243	21.5	12	27.3	255	21.7
Strongly disagree	348	30.8	4	9.1	352	30.0
Mean	3.76		3.27		3.75	

^a^Excluding those unsure about plans.

^b^Independent *T* test: *t* = 2.961, *p* = 0.003.

^c^Independent *T* test: *t* = 3.230, *p* = 0.001.

Table 3 also shows that 18% (of those who had decided their future plans) agreed that they had a good understanding of how the Irish healthcare system works (*n* = 8) with 36% (*n* = 607) disagreeing or strongly disagreeing. As with training, a significantly larger proportion of those not going abroad agreed or strongly agreed that they understood how the Irish healthcare system worked compared to those definitely or contemplating going or those unsure (18% compared to 8%; Independent *T* test, *t* = 3.230, *p* < 0.001). There were no significant differences in rating understanding of the Irish healthcare system by sex (Independent *T* test, *t* = −1.112, *p* = 0.266). A larger proportion of those aged over 21 years (10%) agreed that they understood how the Irish healthcare system works compared to those aged under 21 years (5%). This pattern was statistically significant (Independent *T* test, *t* = 7.547, *p* < 0.001).

## Discussion

The shortage of medical doctors is an international challenge in both developed and developing countries. The World Health Organisation (WHO) has estimated that there is a worldwide shortage of 2.3 million physicians, nurses, and midwives [22], which is expected to persist in many developing countries [23]. In Ireland, severe shortages of both general practitioners and hospital doctors are forecast to persist into the next decade [11,24]. Our finding suggest that this shortfall is set to continue with over a third of Irish medical students definitely planning to migrate and over half contemplating migrating after their graduation or intern year. These findings are similar to other Irish studies of medical students, NCHDs, and interns which showed the proportion considering emigration ranged from 50%–80% [7,12,15]. This represents a significant loss to the Irish healthcare system, particularly in terms of the costs involved in training medial students, plus the cost of recruiting replacements, and the additional workload pressures if replacements cannot be found.

Our study shows that Irish medical students express similar emigration intentions to medical students in low- and middle-income countries. A study of 240 Indian medical students demonstrated that a high proportion of students (59%) considered leaving India for further training [25]. A study of 425 Lebanese medical students found that 96% intended to travel abroad for specialty training, with only 25% intending to return directly after the completion of the training programme [26]. A similar situation is seen in Pakistan with 65%–95% of final-year medical students intending to emigrate [27]. A study of Polish medical students showed that 62% of respondents planned to seek employment abroad; however, unlike our study, they demonstrated that senior Polish medical students expressed less desire to emigrate [28]. A study of South African nursing and medical students reported that 59% of medical students indicated that they were likely or very likely to emigrate within 5 years of graduation [29]. A common factor in the health systems of all the countries mentioned above is the limited access to postgraduate training and a lack of transparency regarding career progression, including the processes for applying to postgraduate training. This highlights the importance of correcting these issues in the Irish setting.

Studies have shown that enrolling students in postgraduate training may influence their decision to permanently remain in the country in which they received training [30,31]. Although postgraduate training in Ireland has expanded through the development of new training programmes [6,8], our study suggests that this information is not being relayed to students. This is evidenced by the fact that only a third of respondents understand what postgraduate training entails. This is in accordance with a position paper by the Irish Medical Organisation that highlights a lack of clarity in career paths and postgraduate speciality training [32]. We also found an association between students who stated that they do not understand what postgraduate training entails and those who intend to or are considering emigration. It might be that a lack of understanding may lead students to choose to emigrate or, alternatively, that those who choose to emigrate do not seek information on postgraduate training. Although it is not possible to provide a definitive explanation for this pattern, if all medical students were informed about postgraduate training and career opportunities in Ireland, they would be in a better position to make decisions about migrating upon completion of their training. In addition, as the shortage of doctors is so great in Ireland, it is suggested that postgraduate opportunities should also be made more accessible to non-EU students that are trained in Ireland. Current European working laws make it difficult for non-EU graduates of Irish medical schools to obtain Irish intern or pre-registration/foundation-year positions; they are therefore lost to the system immediately upon graduation. The creation of additional training positions for non-EU graduates can be offset in the form of return-of-service arrangements. Return-of-service arrangements are commonplace in Canada and Australia [33], whereby costs of medical training are completely or heavily subsidised in return for service for a specified number of years in training posts in the national healthcare service.

In examining intentions to migrate, we found that there was a greater proportion of respondents definitely planning to migrate at the senior stage (38%) compared to those at the junior stage (28%) of training. However, after controlling for age and gender, intentions to definitely migrate appear strongest at the intermediate stage. This suggests that the early experiences of clinical training in the Irish clinical setting may be exacerbating intentions to migrate. In the final year, some may be reappraising their decision to migrate when final decisions in terms of future plans are more imminent. Initiatives designed to impact on retention and migration patterns must therefore be introduced early in medical training and perhaps repeated in the penultimate and final years to help influence decision making.

Career opportunities were the most frequently given reason influencing respondents’ intentions to emigrate (85%), compared to only 4% in a survey of U.K. junior doctors who cited career prospects [18]. The Irish health system is currently increasing the number of consultant positions which should enhance career opportunities [8]. However, in the U.K., the promise of consultant positions following training has led to concern that there may be an oversupply of hospital consultants in the future [34]. Any workforce planning that increases consultant (senior physician or surgeon) positions in Ireland to enhance career opportunities should be carefully managed to match workforce needs to allow employment opportunities to remain competitive and appealing. Career opportunities could also be provided by developing specialist training programmes that commence directly after graduation, reducing the need for students to look abroad for career opportunities. With the lack of clarity and suboptimal definition of the pathways in postgraduate training in Ireland, there is a potential student perception that time spent abroad is of greater value and may confer an advantage in subsequent career progression.

In our study, 83% of respondents cited working conditions as a reason for migrating, double that found in a U.K. study of qualified doctors [18]. A qualitative study of working conditions of doctors in Ireland describes a number of difficulties experienced including the following: unrealistic workloads, staff shortages, extended working hours, irregular and frequently interrupted breaks, fatigue, being undervalued by the community, insufficient training, and a sense of a lack of power to influence change in the healthcare system [19]. Improving working conditions is clearly vital, particularly as working conditions may be seen to be linked with lifestyle and the standard of training, which were also other key reasons for deciding to emigrate.

Salary was reported as a key factor influencing intentions to migrate (65%). This is supported by studies that show that debt and perceived income also influence the career paths of both medical students and qualified physicians [35,36]. Medical students have a high-debt burden in the undergraduate phase and also incur further substantial debt during training [37]. It is therefore inevitable that salary is an increasingly important migration factor. Compared to their counterparts, junior doctors in Ireland have a higher base pay than those in the U.K. but lower than those in Australia [38-40]. However, when attempting to make any rudimentary analysis of income, consideration must also be given to overtime pay, working hours per week, cost of living, taxation, and job satisfaction, which is beyond the scope of this study.

Lifestyle was cited 15% more than salary as the reason for going abroad. The increased effect of lifestyle on career choices has also been shown in US senior medical students [41]. This suggests that although financial remuneration is a factor, it must also be accompanied by a change to the lifestyle of a junior doctor as depicted by McGowan et al. [19], where they feel underappreciated and undervalued.

Migration for education has increased dramatically in the past decade. While historically this was seen at a postgraduate training level, it is now more commonplace for students to migrate at younger ages and at the undergraduate level [42]. Our study demonstrates that migration is considered at the student level in a developed country, a growing trend seen internationally. While “brain drain” has historically been described in developing countries [17], the ease of movement between countries may result in several developed countries to also experience this “brain drain” phenomenon. Understanding why medical graduates of a developed country would consider migration is therefore crucial in developing policies for their retention.

### Limitations

The 37% response rate achieved by our web-based survey was lower than similar U.K. studies that used postal questionnaires [30]. A higher response rate may have been achieved if the survey had been left open for a longer time period. Web-based surveys have been shown to have lower response rates than postal surveys [43]. Although the study does include a large sample size, the possibility of non-responder bias remains.

As the medical schools administered the survey, it was not possible to determine whether the e-mail lists of registered students were complete and up to date. In addition, this form of administration meant that it was possible for respondents to complete the survey more than once. However, it must be noted that of those who provided an e-mail address to enter the draw (71%), no multiple e-mails were provided.

Our study used location of secondary education as proxy to determine nationality, as citizenship or country of birth alone would not adequately define the demographic we were interested in. It was recognised that many students hold Irish citizenship status even though they would not consider themselves primarily Irish because they were raised abroad or have lived abroad for many years. Similar migration studies have recognised this issue and used location of childhood home as a proxy [30]; however, for the reason alluded to above, we felt that the country in which an individual completed secondary school was better suited for this analysis.

Although factors such as knowledge of what postgraduate training entails is associated with intentions to emigrate, it is unclear whether this lack of knowledge causes people to decide to emigrate or, alternatively, whether those who intend to migrate do not seek such information. This places limitations in terms of the ability to generate specific recommendations in terms of postgraduate training from the study.

To identify the factors influencing decisions to go abroad for work, respondents selected all applicable factors from a pre-determined list. As respondents were not asked to rank these factors, we were not able to identify the key factors influencing decisions. In addition, some of the factors presented to respondents may have been too generic or ambiguous in meaning such as “lifestyle” or “career opportunities”. This limits the ability to prioritise and develop specific policy interventions on the basis of the study findings.

## Conclusion

The Irish healthcare system finds itself in a position with a shortage of qualified doctors and a significant proportion of medical students indicating an intention to leave. The potential future emigration of 88% of Irish national medical students poses an immediate and severe threat to the sustainability of the Irish healthcare service. Urgent interventions are needed at an undergraduate level including providing a better understanding of career structures and the organisation of the health service. Interventions at the postgraduate level may include streamlining specialty training pathways, addressing pay and working conditions in training programmes, and clearer workforce planning for more senior posts, in order to retain medical graduates and entice those that have already emigrated to return.

### Ethical approval

This study was approved by the Research Ethics Committee at the National University of Ireland, Galway (Ref:13/Jan/05).

## Endnote

^a^Personal communication.

## Abbreviations

OECD: Organisation for Economic Cooperation and Development
U.K.: United Kingdom
WHO: World Health Organisation
